# Supplementary material for: Fresnel reflection coefficients in the Fourier domain for a planar surface in uniform motion parallel to its interface
Source: Sci Rep. 2025 Dec 18;15:45755. doi: 10.1038/s41598-025-28668-1 (PMC12756287; doi:10.1038/s41598-025-28668-1)
Supplement: Supplementary file 1 — Supplementary Information. [file 41598_2025_28668_MOESM1_ESM.pdf]

# Supplemental Material for "Fresnel reflection coefficients in the Fourier domain for a planar surface in uniform motion parallel to its interface"

Stéphane Azar[1], Francisco J. Rodríguez-Fortuño[1,*], and Sebastian Golat[1]

[1]Department of Physics and London Centre for Nanotechnology, King's College London, Strand, London WC2R 2LS, UK
[*]francisco.rodriguez_fortuno@kcl.ac.uk

August 18, 2025

## A Boost of Jones vector in an arbitrary direction

In the main text, we considered boosts along the $x$-direction. In this appendix we consider boosts along any arbitrary direction, requiring an understanding of how the Jones vector of polarisation transforms for any plane or evanescent wave under arbitrary Lorentz boosts. This appendix formalises that transformation.

Consider the action of a Lorentz boost on the components of 4-vector $k^\mu$, which can be represented as a matrix:

$$k'^\mu = \begin{pmatrix} k_0' \\ \mathbf{k}' \end{pmatrix} = \begin{pmatrix} \gamma & -\gamma\boldsymbol{\beta}^\intercal \\ -\gamma\boldsymbol{\beta} & \boldsymbol{\Gamma}_\parallel(\boldsymbol{\beta}) \end{pmatrix} \begin{pmatrix} k_0 \\ \mathbf{k} \end{pmatrix} = \Lambda^\mu{}_\nu k^\nu \tag{1}$$

where $\boldsymbol{\beta} = \beta\hat{\boldsymbol{\beta}}$ is the relative velocity of the boost, with $\beta = v/c$ and $\hat{\boldsymbol{\beta}}$ being the direction of the boost, and we have defined a parallel stretching operator

$$\boldsymbol{\Gamma}_\parallel(\boldsymbol{\beta}) = (\hat{I} - \hat{\boldsymbol{\beta}}\hat{\boldsymbol{\beta}}^\intercal) + \gamma\hat{\boldsymbol{\beta}}\hat{\boldsymbol{\beta}}^\intercal = \boldsymbol{P}_\perp(\hat{\boldsymbol{\beta}}) + \gamma\boldsymbol{P}_\parallel(\hat{\boldsymbol{\beta}})\,, \tag{2}$$

where $\hat{I} = \mathrm{diag}(1,1,1)$, and $\boldsymbol{P}_\perp$ and $\boldsymbol{P}_\parallel$ are transverse and longitudinal projection operators. This transformation works for the frequency and wavevector, however, the electromagnetic field is described by an antisymmetric rank-2 tensor, which transforms in the following way:

$$F'^\mu{}_\nu = \Lambda^\mu{}_\rho F^\rho{}_\sigma \Lambda^\sigma{}_\nu = (\Lambda F \Lambda^{-1})^\mu{}_\nu = (\mathrm{Ad}_\Lambda F)^\mu{}_\nu \tag{3}$$

where $\mathrm{Ad}_\Lambda(X) = \Lambda X \Lambda^{-1}$ is called the adjoint representation of the Lorentz group. Since $F^{\mu\nu}$ is an antisymmetric 4-by-4 matrix with six independent components, it is isomorphic to a 6-vector $\big(\mathbf{E}/c, \mathbf{B}\big)^\intercal$ and the adjoint representation $\mathrm{Ad}_\Lambda$ can then be understood as a 6-by-6 matrix acting as follows:

$$\vec{F}' = \begin{pmatrix} \mathbf{E}'/c \\ \mathbf{B}' \end{pmatrix} = \begin{pmatrix} \boldsymbol{\Gamma}_\perp(\boldsymbol{\beta}) & \gamma\boldsymbol{\beta}\times \\ -\gamma\boldsymbol{\beta}\times & \boldsymbol{\Gamma}_\perp(\boldsymbol{\beta}) \end{pmatrix} \begin{pmatrix} \mathbf{E}/c \\ \mathbf{B} \end{pmatrix} = \mathrm{Ad}_\Lambda \vec{F}\,, \tag{4}$$

where we use arrows for 6-vectors, and we introduced the transverse stretching operator defined as:

$$\mathbf{\Gamma}_\perp(\boldsymbol{\beta}) = \gamma(\hat{I} - \hat{\boldsymbol{\beta}}\hat{\boldsymbol{\beta}}^\intercal) + \hat{\boldsymbol{\beta}}\hat{\boldsymbol{\beta}}^\intercal = \gamma \boldsymbol{P}_\perp(\hat{\boldsymbol{\beta}}) + \boldsymbol{P}_\parallel(\hat{\boldsymbol{\beta}})\,. \tag{5}$$

Notice that these 6-vectors can be understood as 2-vectors (separate electric and magnetic field) with components that are themselves 3-vectors (their spatial components), and these can be treated separately.[1] This also means that one can write the matrix $\mathrm{Ad}_\Lambda$ in terms of the tensor product of Pauli matrices acting on the 2-vectors (separating **E** and **H** contributions) and 3-by-3 matrices acting on 3-vector components (so $\hat{\mathbf{x}}$, $\hat{\mathbf{y}}$ ,$\hat{\mathbf{z}}$ components):

$$\mathrm{Ad}_\Lambda(\boldsymbol{\beta}) = \mathbf{\Gamma}_\perp(\boldsymbol{\beta}) \begin{pmatrix} 1 & 0 \\ 0 & 1 \end{pmatrix} + \mathrm{i}\gamma\boldsymbol{\beta}\times \begin{pmatrix} 0 & -\mathrm{i} \\ \mathrm{i} & 0 \end{pmatrix} = \mathbf{\Gamma}_\perp(\boldsymbol{\beta})\boldsymbol{\sigma}_0 + \mathrm{i}\gamma\boldsymbol{\beta}\times\boldsymbol{\sigma}_2\,, \tag{6}$$

where we keep the tensor product implicit.[2] The plane wave fields eq. (1) can be written in this representation as:

$$c\vec{F}(t,\mathbf{r}) = \begin{pmatrix} \mathbf{E}(t,\mathbf{r}) \\ c\mathbf{B}(t,\mathbf{r}) \end{pmatrix} = \underbrace{\begin{pmatrix} A_s\hat{\mathbf{e}}_s + A_p\hat{\mathbf{e}}_p \\ A_p\hat{\mathbf{e}}_s - A_s\hat{\mathbf{e}}_p \end{pmatrix}}_{c\vec{F}_0(\omega,\mathbf{k})} \mathrm{e}^{\mathrm{i}\mathbf{k}\cdot\mathbf{r}-\mathrm{i}\omega t} = \underbrace{\begin{pmatrix} \hat{\mathbf{e}}_s & \hat{\mathbf{e}}_p \\ -\hat{\mathbf{e}}_p & \hat{\mathbf{e}}_s \end{pmatrix}}_{\boldsymbol{M}(\omega,\mathbf{k})} \underbrace{\begin{pmatrix} A_s \\ A_p \end{pmatrix}}_{\boldsymbol{\mathcal{J}}} \mathrm{e}^{\mathrm{i}\mathbf{k}\cdot\mathbf{r}-\mathrm{i}\omega t}\,, \tag{7}$$

where in the last step, we wrote it in terms of the familiar Jones vector of polarisation $\boldsymbol{\mathcal{J}}$ (using bold calligraphic to emphasise it is a 2-vector). The linear map $\boldsymbol{M} : \mathbb{C}^2 \to \mathbb{C}^6$ such that $\boldsymbol{\mathcal{J}} \mapsto c\vec{F}_0(\omega,\mathbf{k}) = \boldsymbol{M}(\omega,\mathbf{k})\boldsymbol{\mathcal{J}}$ might seem a bit strange at first, but it is well defined. For instance, it can be represented by a rectangular matrix:

$$\boldsymbol{M}\boldsymbol{\mathcal{J}} = \underbrace{\begin{pmatrix} \hat{\mathbf{e}}_s & \hat{\mathbf{e}}_p \\ -\hat{\mathbf{e}}_p & \hat{\mathbf{e}}_s \end{pmatrix}}_{6\times 2} \underbrace{\begin{pmatrix} A_s \\ A_p \end{pmatrix}}_{2\times 1} = \begin{pmatrix} \hat{\mathbf{x}}\cdot\hat{\mathbf{e}}_s & \hat{\mathbf{x}}\cdot\hat{\mathbf{e}}_p \\ \hat{\mathbf{y}}\cdot\hat{\mathbf{e}}_s & \hat{\mathbf{y}}\cdot\hat{\mathbf{e}}_p \\ \hat{\mathbf{z}}\cdot\hat{\mathbf{e}}_s & \hat{\mathbf{z}}\cdot\hat{\mathbf{e}}_p \\ -\hat{\mathbf{x}}\cdot\hat{\mathbf{e}}_p & \hat{\mathbf{x}}\cdot\hat{\mathbf{e}}_s \\ -\hat{\mathbf{y}}\cdot\hat{\mathbf{e}}_p & \hat{\mathbf{y}}\cdot\hat{\mathbf{e}}_s \\ -\hat{\mathbf{z}}\cdot\hat{\mathbf{e}}_p & \hat{\mathbf{z}}\cdot\hat{\mathbf{e}}_s \end{pmatrix} \begin{pmatrix} A_s \\ A_p \end{pmatrix}\,. \tag{8}$$

This map can again be written in terms of Pauli matrices as:

$$\begin{pmatrix} \hat{\mathbf{e}}_s & \hat{\mathbf{e}}_p \\ -\hat{\mathbf{e}}_p & \hat{\mathbf{e}}_s \end{pmatrix} = \hat{\mathbf{e}}_s \begin{pmatrix} 1 & 0 \\ 0 & 1 \end{pmatrix} + \mathrm{i}\hat{\mathbf{e}}_p \begin{pmatrix} 0 & -\mathrm{i} \\ \mathrm{i} & 0 \end{pmatrix} = \hat{\mathbf{e}}_s\boldsymbol{\sigma}_0 + \mathrm{i}\hat{\mathbf{e}}_p\boldsymbol{\sigma}_2\,. \tag{9}$$

We can define a left inverse of $\boldsymbol{M}$ by requiring $\boldsymbol{M}_L\boldsymbol{M} = \boldsymbol{\sigma}_0$ which is satisfied, for example, by:

$$\boldsymbol{M}_L\boldsymbol{M} = \underbrace{\begin{pmatrix} \hat{\mathbf{e}}_s^\intercal & 0 \\ 0 & \hat{\mathbf{e}}_s^\intercal \end{pmatrix}}_{2\times 6} \underbrace{\begin{pmatrix} \hat{\mathbf{e}}_s & \hat{\mathbf{e}}_p \\ -\hat{\mathbf{e}}_p & \hat{\mathbf{e}}_s \end{pmatrix}}_{6\times 2} = \underbrace{\begin{pmatrix} 1 & 0 \\ 0 & 1 \end{pmatrix}}_{2\times 2} = \boldsymbol{\sigma}_0\,, \quad \text{since} \quad \hat{\mathbf{e}}_s^\intercal\hat{\mathbf{e}}_s = 1, \text{ and } \hat{\mathbf{e}}_s^\intercal\hat{\mathbf{e}}_p = 0\,, \tag{10}$$

[1]This is because they live in a vector space $\mathbb{R}^6 = \mathbb{R}^2 \otimes \mathbb{R}^3$ (or $\mathbb{C}^6 = \mathbb{C}^2 \otimes \mathbb{C}^3$ for analytic signals or phasors).

[2]The symbol $\boldsymbol{\beta}\times$ denotes the antisymmetric matrix representing the cross product operator. That is, $\boldsymbol{\beta}\times\mathbf{a} \equiv [\boldsymbol{\beta}\times]\,\mathbf{a}$ for any vector $\mathbf{a}$, where $[\boldsymbol{\beta}\times]$ is the $3\times 3$ matrix:

$$[\boldsymbol{\beta}\times] = \begin{pmatrix} 0 & -\beta_z & \beta_y \\ \beta_z & 0 & -\beta_x \\ -\beta_y & \beta_x & 0 \end{pmatrix}.$$

notice that there is not one unique inverse (a well-known feature of one-sided inverses), but infinitely many, as

$$[\boldsymbol{M}_L + \boldsymbol{A}(\boldsymbol{I} - \boldsymbol{M}\boldsymbol{M}_L)]\boldsymbol{M} = \boldsymbol{M}_L\boldsymbol{M} + \boldsymbol{A}(\boldsymbol{M} - \boldsymbol{M}) = \boldsymbol{\sigma}_0\,, \quad \forall \boldsymbol{A} \in \mathbb{C}^{2\times 6}\,,\ \boldsymbol{I} = \mathrm{diag}(1,1,1,1,1,1)\,. \tag{11}$$

Using any of these inverses, we can project the electromagnetic bivector for any plane wave (or evanescent wave) onto the Jones vector as follows:

$$\boldsymbol{\mathcal{J}} = \boldsymbol{M}_L(\omega, \mathbf{k})c\vec{F}_0(\omega, \mathbf{k})\,, \tag{12}$$

Now suppose that we want to know $\boldsymbol{\mathcal{J}}'$ in a boosted frame, we can write

$$\boldsymbol{\mathcal{J}}' = \boldsymbol{M}'_L(\omega', \mathbf{k}')\,\mathrm{Ad}_\Lambda(\boldsymbol{\beta})c\vec{F}_0(\omega, \mathbf{k}) = \boldsymbol{M}'_L(\omega', \mathbf{k}')\,\mathrm{Ad}_\Lambda(\boldsymbol{\beta})\boldsymbol{M}(\omega, \mathbf{k})\boldsymbol{\mathcal{J}} = \boldsymbol{\Lambda}(\omega, \mathbf{k}, \boldsymbol{\beta})\boldsymbol{\mathcal{J}}\,, \tag{13}$$

where $\boldsymbol{\Lambda}(\omega, \mathbf{k}, \boldsymbol{\beta})$ is how the Lorentz boost is represented on the Jones vector for any electromagnetic field with a $\mathrm{e}^{\mathrm{i}\mathbf{k}\cdot\mathbf{r}-\mathrm{i}\omega t}$ dependence, including plane waves and evanescent waves, accounting for the fact that the polarisation basis is different in each frame. We can use the eqs. (6) and (10) to write the most general expression for the boost of the Jones vector explicitly in terms of polarisation basis vectors as (choosing $\boldsymbol{M}'_L = \hat{\mathbf{e}}_s'^{\mathsf{T}}\boldsymbol{\sigma}_0$):

$$\boldsymbol{\Lambda}(\omega, \mathbf{k}, \boldsymbol{\beta}) = \hat{\mathbf{e}}'_s \cdot [\boldsymbol{\Gamma}_\perp \hat{\mathbf{e}}_s - \gamma\boldsymbol{\beta} \times \hat{\mathbf{e}}_p]\boldsymbol{\sigma}_0 + \hat{\mathbf{e}}'_s \cdot [\boldsymbol{\Gamma}_\perp \hat{\mathbf{e}}_p + \gamma\boldsymbol{\beta} \times \hat{\mathbf{e}}_s]\mathrm{i}\boldsymbol{\sigma}_2\,. \tag{14}$$

For a wavevector $\mathbf{k}^\pm = k_x\hat{\mathbf{x}} + k_y\hat{\mathbf{y}} \pm k_z\hat{\mathbf{z}}$ with the $s$ and $p$ polarisation basis vectors defined in general as:

$$\hat{\mathbf{e}}_s = \frac{\hat{\mathbf{z}} \times \mathbf{k}_t}{k_t} = \frac{k_x\hat{\mathbf{y}} - k_y\hat{\mathbf{x}}}{k_t}\,, \quad \hat{\mathbf{e}}_p^\pm = \frac{\pm\mathbf{k}_t k_z - k_t^2\hat{\mathbf{z}}}{k_t k_0} = \frac{\pm(k_x\hat{\mathbf{x}} + k_y\hat{\mathbf{y}})k_z - k_t^2\hat{\mathbf{z}}}{k_t k_0}\,, \tag{15}$$

we can find the diagonal $\boldsymbol{\sigma}_0$ terms for an arbitrary boost $\boldsymbol{\beta} = \beta_x\hat{\mathbf{x}} + \beta_y\hat{\mathbf{y}} + \beta_z\hat{\mathbf{z}}$ to be (after some algebra):

$$\hat{\mathbf{e}}'_s \cdot [\boldsymbol{\Gamma}_\perp \hat{\mathbf{e}}_s - \gamma\boldsymbol{\beta} \times \hat{\mathbf{e}}_p^\pm] = \frac{\mathbf{k}'_t}{k'_t} \cdot \left[\frac{\mathbf{k}'_t}{k_t} + \frac{\gamma k_z}{k_t k_0}(\boldsymbol{\beta}_t k_z \mp \beta_z \mathbf{k}_t)\right] = \frac{k'_t}{k_t} + \frac{k_z}{k_0}\frac{\gamma(\boldsymbol{\beta}k_z \mp \beta_z\mathbf{k}_t)\cdot\mathbf{k}'_t}{k_t k'_t}\,. \tag{16}$$

Notice that $(\hat{\boldsymbol{\beta}} \cdot \mathbf{k}'_t)$ is the part of $\mathbf{k}'_t$ along the boost direction, so if $\hat{\boldsymbol{\beta}} = \hat{\mathbf{x}}$ we obtain:

$$\hat{\mathbf{e}}'_s \cdot [\boldsymbol{\Gamma}_\perp \hat{\mathbf{e}}_s - \gamma\beta\hat{\mathbf{x}} \times \hat{\mathbf{e}}_p^\pm] = \frac{k'_t}{k_t} + \gamma\beta\frac{k'_x k_z^2}{k'_t k_t k_0}\,, \tag{17}$$

which is precisely the diagonal terms in eq. (9) in the main text. The antisymmetric mixed terms (with $\mathrm{i}\boldsymbol{\sigma}_2$) are

$$\hat{\mathbf{e}}'_s \cdot [\boldsymbol{\Gamma}_\perp \hat{\mathbf{e}}_p^\pm + \gamma\boldsymbol{\beta} \times \hat{\mathbf{e}}_s] = \frac{(\hat{\mathbf{z}} \times \hat{\boldsymbol{\beta}})\cdot\mathbf{k}_t}{k'_t k_t}\left[\frac{\mp\gamma\beta k'_0 k_z}{k_0} + \frac{\beta_z(\gamma-1)}{\beta}\left(\gamma\boldsymbol{\beta}\cdot\mathbf{k}'_t + (1+\gamma)k'_0 - \frac{k_t^2}{k_0}\right)\right] \tag{18}$$

where $(\hat{\mathbf{z}} \times \hat{\boldsymbol{\beta}})\cdot\mathbf{k}_t = (\hat{\mathbf{z}} \times \hat{\boldsymbol{\beta}})\cdot\mathbf{k}'_t$ is the part of $\mathbf{k}_t$ that is both transverse to $\hat{\boldsymbol{\beta}}$ as well as $\hat{\mathbf{z}}$, hence for $\hat{\boldsymbol{\beta}} = \hat{\mathbf{x}}$:

$$\hat{\mathbf{e}}'_s \cdot [\boldsymbol{\Gamma}_\perp \hat{\mathbf{e}}_p^\pm + \gamma\boldsymbol{\beta} \times \hat{\mathbf{e}}_s] = \mp\gamma\beta\frac{k'_0 k_z k_y}{k_0 k'_t k_t} \tag{19}$$

which matches the antisymmetric part in eq. (9) in the main text.
